# What I Know about Phytolacca

**Published:** 1884-12

**Authors:** Wm. Jefferson Guernsey

**Affiliations:** Phila.


### WHAT I KNOW ABOUT PHYTOLACCA.

WM. JEFFERSON GUERNSEY, M. D., PHILA.

It is but little, yet that knowledge, meagre as it is, has not only proven to the writer how potent is *the* law of cure, but given much relief from suffering hard, indeed, to be endured.

Who can paint a picture of greater mental agony than that of a poor, unmanned man, who, having once endured the tortures of quinsy, again feels the grip of this visionary hangman? who with horror recalls the nearly locked jaws, the swollen tongue, the breath that was fetid beyond endurance, even to himself, the constant and profuse flow of saliva that compelled frequent efforts at deglutition, the bare thought of which was agony; the sleepy sleeplessness, the restlessness, debility, the starving hunger, with loathing of food?—to such a "hell on earth" does he look forward with fear and trembling, yet with utmost certainty. What, then, must be the relief when that great burden of fear is removed by being assured that his disease can be arrested!

Homœopathy here scores one of its triumphs; for not only does it abort this painful affection, but by so doing it eventually destroys totally the liability of its recurrence. Scarcely a case of tonsilitis but can be at once resolved if prescribed for early.

We have many remedies capable of doing this, but none has served me more faithfully than Phytolacca decandra. Unless some other remedy *is indicated*, I usually think of that. It is especially useful if the patient complain of pain at the root of the tongue or to the ears when swallowing, of much dryness of the throat with the soreness, and the fauces and tonsils appear dark—perhaps of a bluish cast. Very many times has one prescription of one, two, or never more than three powders of the 50 M of this remedy, an hour apart (when not half so well indicated), been all the medicine used, and generally afforded relief in a few hours, or at most a day. A case so treated will not *trouble* you often.

One lady, who had *expected* this semi-annual visitor and never been disappointed in a dozen years, was cured the first time thus in twenty-four hours, the second in twelve hours, and has had an entire immunity from it now for five years. This patient so lauded my praises once in a store where I chanced to meet her that I was glad to make my escape. Yet not I, but Homœopathy, deserved all the glory.

We have looked with a pitying eye on the quinsy patient. What shall we say—what need be said—of the miserable being with a "gathered breast"? Where is there a merciless nurse who will not shudder at the thought? Phytolacca, again, may save many a long, feverish night, many a bitter hour of suffering, and many a heartache, as the mother thinks of her little one. The right breast is the one affected; the gland seems full to overflowing and has, perhaps, for several days yielded an over-abundance of milk, even to the extent of prostrating the patient; the breast feels stony, hard, and painful; she is totally indifferent to life or predicts her death. Again, the breast has been abscessed and badly treated; large, gaping, and inflamed ulcers are seen, having a thin, fetid discharge.

The use of this remedy in mammary troubles is not confined to educated homœopathicians; the "*cow doctor*" knew something about its virtues before you or I dreamed of "similia similibus curantur."

About two years ago a tall fellow, of splendid physique, limped into my office with what he had been told was "sky attic." The pain was worse in, and almost wholly confined to, the right limb, aggravated at night, and had a downward course.

℞. Four powders of Phytol.$^{50m}$, and in twenty-four hours he walked without limping and had slept nearly all night without pain. This patient had taken the usual "hundreds of dollars' worth of medicines" and suffered for years. The indications in his case were "clean cut" for the remedy—hence the quick relief.

Not much space has been awarded Phytolacca in the therapeutical works of Homœopathy, and he who trusts to these alone in preference to the more laborious but safer plan of "symptom hunting" in the repertory will lose many a valued suggestion and pet indication of his "grave and reverend seniors." It is a good plan to search the repertory while prescribing, even when apparently sure of the remedy. It often leads to a change of base in treatment, and several times has the writer found the subject of this little paper thrust unexpectedly upon him.

